# The complete chloroplast genome of *eriochloa villosa* (thunb.) kunth

**DOI:** 10.1080/23802359.2025.2519217

**Published:** 2025-06-24

**Authors:** Haijun Cheng, Yujie Huang, Mei Li, Longjiang Fan, Jian Li

**Affiliations:** a Institute of Crop Sciences & Institute of Bioinformatics, Zhejiang University, Hangzhou, China; b Shandong (Linyi) of Modern Agriculture, Zhejiang University, Linyi, China; c Weed Science Department, Institute of Plant Protection, Shandong Academy of Agricultural Sciences, Jinan, China

**Keywords:** Chloroplast genome, eriochloa villosa, weed, phylogenetic analysis

## Abstract

*Eriochloa villosa* (Thunb.) Kunth, 1829 (Woolly cupgrass), an invasive annual weed in the Poaceae family, poses a significant threat to corn and soybean crop production, resulting in substantial yield reduction. Despite its agronomic importance, genomic resources for this species remain limited. In this study, we report the first complete chloroplast genome assembly of a hexaploid *E. villosa*, which spans 139,777 base pairs and contains 127 annotated genes. This comprehensive chloroplast genomic characterization provides essential foundational resources for further genetic and evolutionary studies within the *Eriochloa* genus.

## Introduction

**Figure 1.**
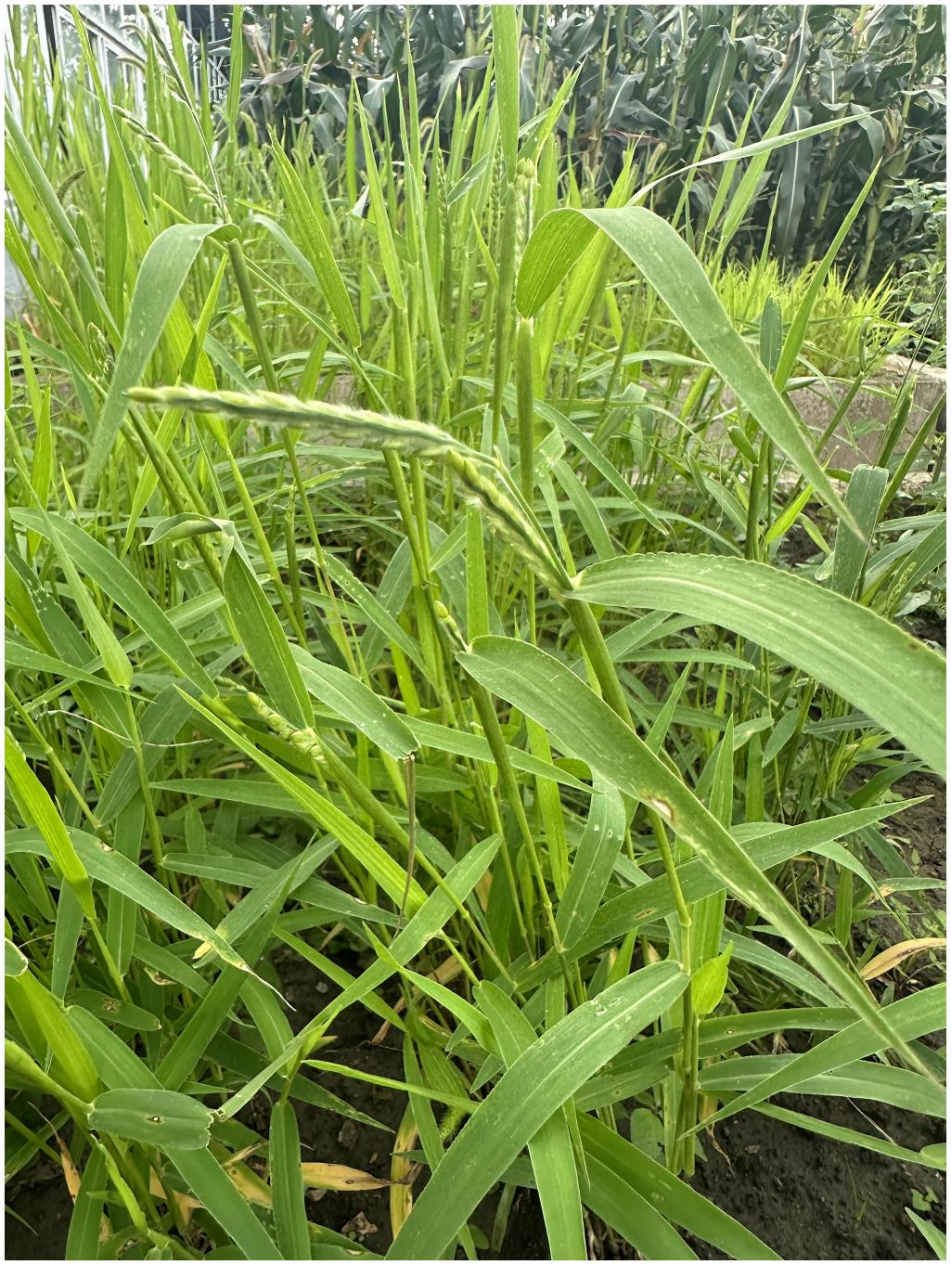
Reference image of *eriochloa villosa* in its natural habitat, taken by corresponding author Jian Li.

*Eriochloa villosa* (Thunb.) Kunth, 1829 (Woolly cupgrass) is an aggressive weed with a flowering and fruiting period from July to October (Figure 1). It is mainly distributed in the northeast, central, and southwest regions of China, with populations also found in Japan, India, the United States, Eastern Europe, and Australia. This species commonly inhabits dry agricultural fields, particularly those cultivated for soybeans and corn (Simard et al. 2015). In severe cases, *E. villosa* can reduce crop yields by nearly 70% through its vigorous axillary branching, shoot production, and tillering capabilities that suppress crop growth (Darbyshire et al. 2003; Han et al. 2023).

The chloroplast genome of angiosperms is notably conserved compared to its nuclear and mitochondrial counterparts, making it an effective resource for examining chloroplast gene function, studying population genetics, and analyzing phylogenetic evolution (Daniell et al. 2016). Moreover, complete chloroplast genome sequences have played a significant role in clarifying some unresolved taxonomic questions in plant systematics (Ruhsam et al. 2015). Here, we sequenced and characterized the complete chloroplast genome of *E. villosa*, providing critical insights into its genomic architecture and evolutionary history.

## Materials and methods

In this research, *E. villosa* specimen was sourced by Haijun Cheng from Changtu, Liaoning Province, China (124.05° E, 42.91° N), and taxonomically verified by Researcher Jian Li from the Plant Protection Institute of the Shandong Academy of Agricultural Sciences. The specimen was deposited in the Herbarium of Zhejiang University under voucher number HZU60147678 (Haijun Cheng, 1978133454@qq.com). Total DNA was used to construct libraries with an average insert size of 350 base pairs, which were sequenced on an Illumina NovaSeq 6000 platform. The sequencing process yielded approximately 52 GB of raw data with paired-end read lengths of 150 bp. Raw reads were quality-filtered using fastp v0.23.4 (Chen 2023) before de novo assembly with NOVOPlasty v4.3.5 (Dierckxsens et al. 2017), using the complete chloroplast genome of the closely related species *Panicum virgatum* (NCBI accession number NC_015990) as a reference. The assembled genome was annotated using GeSeq (Tillich et al. 2017) and visualized with OGDRAW (Greiner et al. 2019). The complete annotated sequence has been deposited in GenBank under accession number PQ238362.

To elucidate the phylogenetic relationships of *E. villosa*, a phylogenetic tree was constructed based on chloroplast genome sequences from six genera: *Alloteropsis*, *Panicum*, *Eriochloa*, *Setaria*, *Echinochloa*, and *Digitaria*, with *Oryza sativa* serving as an outgroup. Sequence alignment was performed using MAFFT v7.525 (Katoh et al. 2002) with the ‘auto’ strategy. A maximum-likelihood phylogenetic tree was constructed using IQ-TREE v2.3.6 (Nguyen et al. 2015) with the recommended settings ‘-m MFP -bb 1000 –bnni.’ The resulting tree was visualized and refined using the online iTOL tool (Letunic and Bork 2019).

## Results

**Figure 2.**
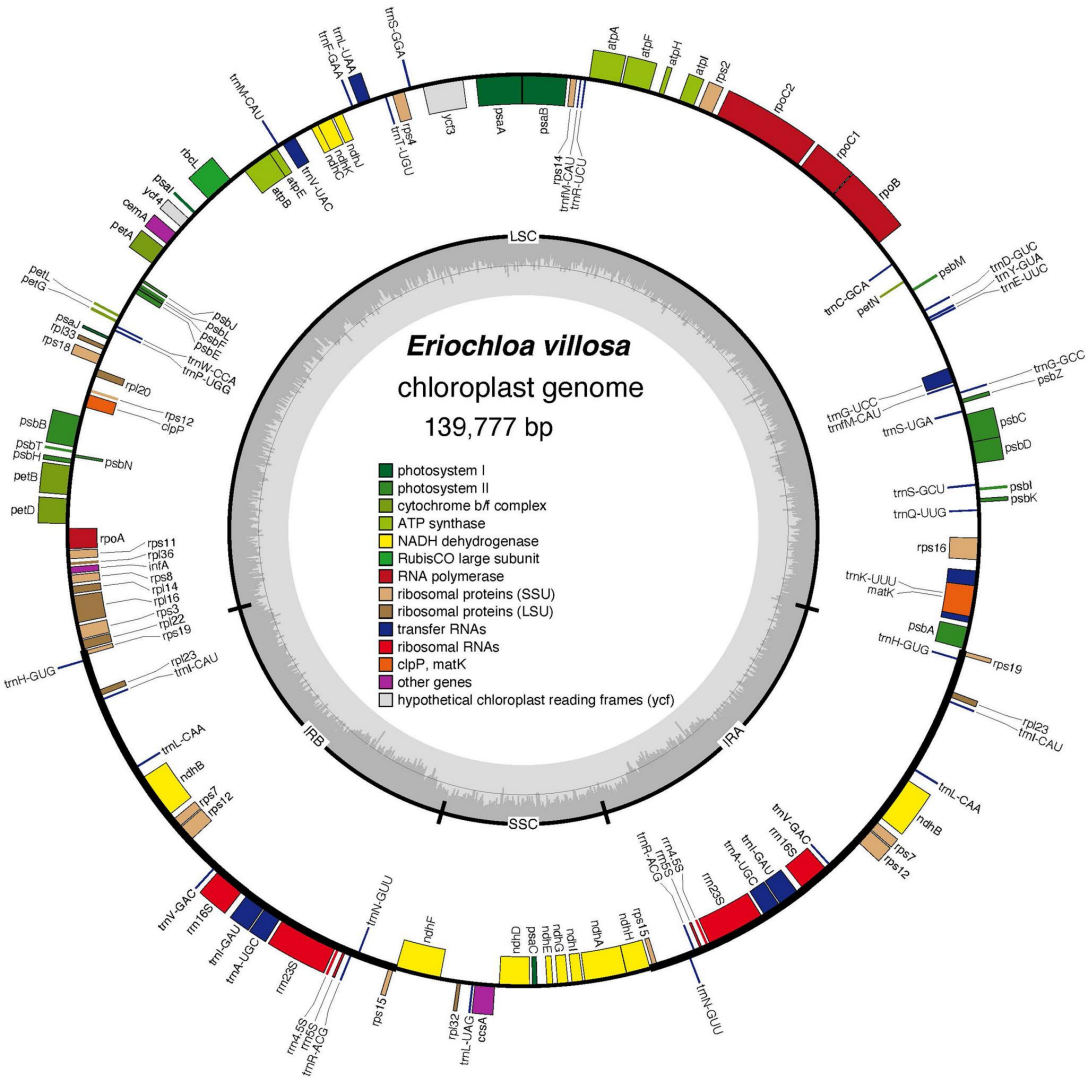
Chloroplast genome map of *eriochloa villosa*. Genes are displayed on the interior and exterior of the circle, with transcription directions indicated as clockwise and counterclockwise. Different colors represent the functional categories of each gene. The black inner circle depicts the chloroplast structure, divided into four regions: the small single-copy region (SSC), large single-copy region (LSC), and two inverted repeats (IRa and IRb). The gray band denotes the GC content throughout the genome.

We successfully assembled the complete 139,777 bp chloroplast genome of *E. villosa* with high precision, achieving an average read depth of 12,878.62× across the entire sequence (Supplementary Figure 1). The genome has a typical quadripartite architecture similar to most angiosperm chloroplasts (Hu et al. 2022), comprising two identical inverted repeat regions (IRa and IRb, each 22,732 bp), a large single-copy region (LSC) of 81,767 bp, and a small single-copy region (SSC) of 12,546 bp (Figure 2). The GC content varies across the regions, with 44.0% in the IR, 36.4% in the LSC, and 32.9% in the SSC.

**Figure 3.**
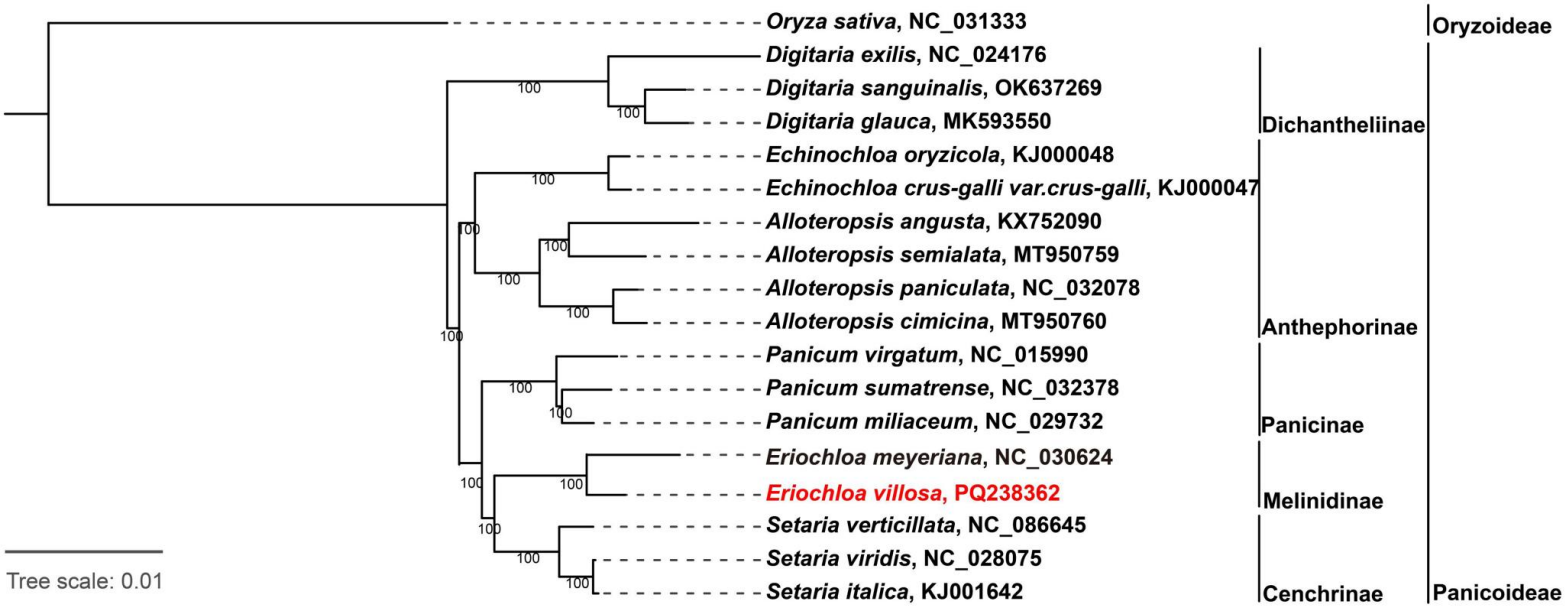
Maximum-Likelihood (ML) phylogenetic tree of 17 species in the poaceae family derived from sequences of complete chloroplast genome (*digitaria exilis*, NC_024176 (Dong et al. 2021); *digitaria sanguinalis*, OK637269; *digitaria glauca*, MK593550 (Bianconi et al. 2020); *echinochloa oryzicola*, KJ000048 (Ye et al. 2014); *echinochloa crus-galli var.crus-galli*, KJ000047 (Ye et al. 2014); *alloteropsis angusta*, KX752090 (Olofsson et al. 2016); *alloteropsis semialata*, MT950759; *alloteropsis paniculata*, NC_032078 (Olofsson et al. 2016); *alloteropsis cimicina*, MT950760; *panicum virgatum*, NC_015990 (Young et al. 2011); *panicum sumatrense*, NC_032378; *panicum miliaceum*, NC_029732 (Cao et al. 2017); *eriochloa meyeriana,* NC_030624; *eriochloa villosa*, PQ238362; *setaria verticillata*, NC_086645; *setaria viridis*, NC_028075 (Wang and Gao 2016a); *setaria italica*, KJ001642 (Wang and Gao 2016b); *Oryza sativa*, NC_031333 as an out group). Bootstrap support value from 1000 replicates is shown on each node.

We identified 127 genes in the complete genome, comprising 81 protein-coding genes, 38 tRNA genes and 8 rRNA genes. Each inverted repeat (IR) region contains an identical set of 18 genes, including 6 protein-coding genes (rps19, rpl23, ndhB, rps7, rps12, rps15), 8 tRNA genes (trnH-GUG, trnI-CAU, trnL-CAA, trnV-GAC, trnL-CAU, trnA-UGC, trnR-AUG, trnN-GUU), and 4 rRNA genes (rrn16S, rrn23S, rrn4.5S, rrn5S). Both cis- and trans-splicing gene structures were verified and annotated using CPGview (Liu et al. 2023), as shown in Supplementary Figures 2 and 3. Eight cis-splicing genes (rps16, atpF, petB, petD, rpl16, ndhA, two copies of ndhB) contained a single intron, whereas the ycf3 gene contained two introns. In addition, one trans-splicing gene (rps12) was identified, producing two transcript types. A phylogenetic tree was constructed using 18 Poaceae species, comprising 17 Panicoideae representatives and one Oryzoideae species as an outgroup. The resulting topology (Figure 3) revealed that all 17 Panicoideae accessions clustered together, forming five distinct clades corresponding to the subtribes Dichanthellinae, Anthephorinae, Panicinae, Melinidinae, and Cenchrinae. Two *Eriochloa* species formed a monophyletic clade that constituted a sister group to *Setaria* with maximum bootstrap support (100%). Phylogenetic relationships among other Poaceae representatives were consistent with previous findings (Jiang et al. 2021).

## Discussion and conclusion

In this study, we successfully assembled and annotated the entire chloroplast genome of a hexaploid *E. villosa* for the first time. The high-quality genome assembly was confirmed by uniform read coverage with an average depth of 12,878.62× across all genomic regions. The chloroplast genome has a typical quadripartite structure (one SSC, one LSC, identical inverted IRa and IRb), which is common for chloroplast genomes among various eukaryotic species (Jiang et al. 2021). The highly conserved GC content, genome size, and structure of chloroplast genomes across Paniceae species underscore the critical evolutionary importance of chloroplasts for photosynthesis during plant diversification (Hu et al. 2022). Phylogenetic analysis revealed that the *Eriochloa* genus is closely related to *Setaria* genus, which further clarified the taxonomic classification of E. villosa within the Poaceae family. The chloroplast genome contains regions that are conserved within species yet divergent between species, resulting in high phylogenetic resolution with maximum bootstrap values (100%), which confirm the reliability of the phylogenetic tree. In addition, this comprehensive chloroplast genome characterization of *E. villosa* provides a foundation for future genetic investigations within the *Eriochloa* genus. Our ongoing research aims to sequence the nuclear genome of *E. villosa* and conduct comparative genomic analyses among the *Eriochloa*, *Melinis*, and *Megathyrsus* genera within the Melinidinae subtribe of Paniceae. Additionally, integration of chloroplast genome data with planned nuclear genome sequence will provide comprehensive insights into the genetic determinants underlying *E. villosa*’s invasive capacity, thereby informing effective and sustainable management strategies for this agriculturally significant weed species.

## Supplementary Material

Supplementary Figure 1.jpg

Supplementary Figure 2.jpg

Supplymentary Figure legend.docx

Supplementary Figure 3.jpg

## Data Availability

The genome sequence data that support the findings of this study are openly available in GenBank of NCBI (https://www.ncbi.nlm.nih.gov/) under the accession number PQ238362, and the associated BioProject, SRA, and Bio-Sample numbers are PRJNA1149314, SRR30285156, and SAMN43241665 respectively.
