# Supplementary figures and images for: The complete chloroplast genome of eriochloa villosa (thunb.) kunth
Source: Mitochondrial DNA B Resour. 2025 Jun 24;10(7):637–40. doi: 10.1080/23802359.2025.2519217 (PMC12207762; doi:10.1080/23802359.2025.2519217)

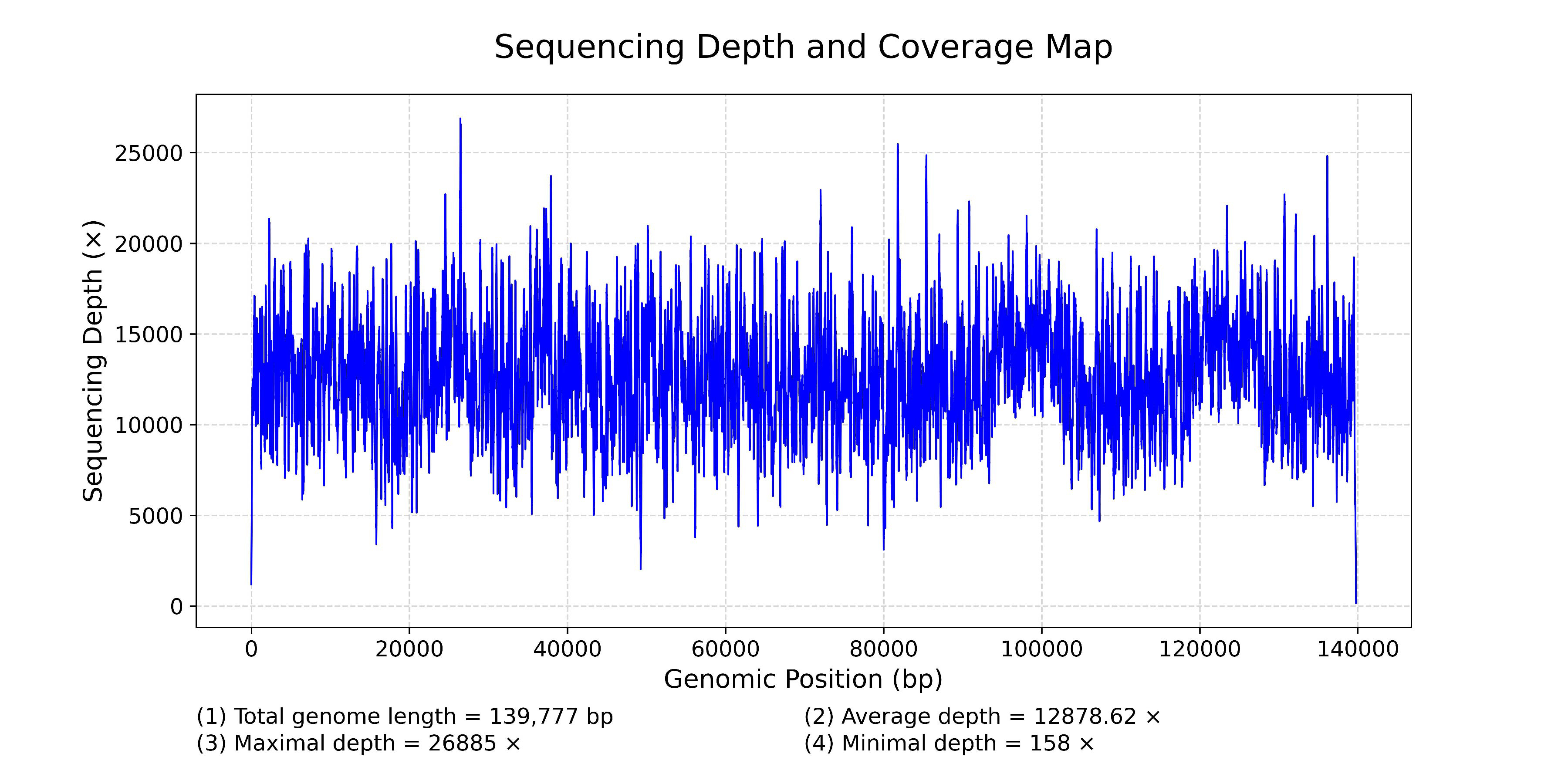

Supplement: Supplementary Figure 1.jpg [file TMDN_A_2519217_SM5726.jpg]

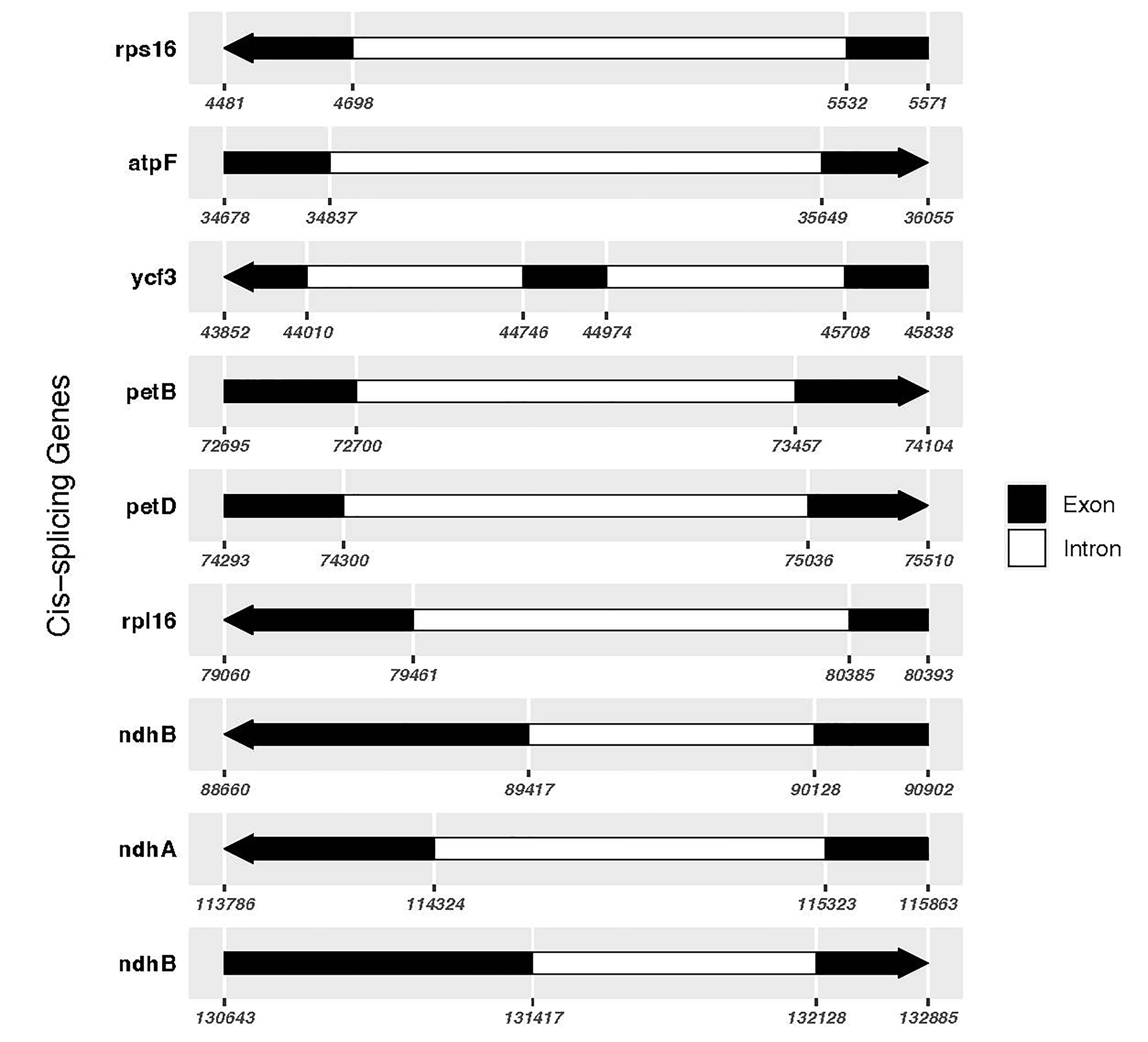

Supplement: Supplementary Figure 2.jpg [file TMDN_A_2519217_SM5725.jpg]

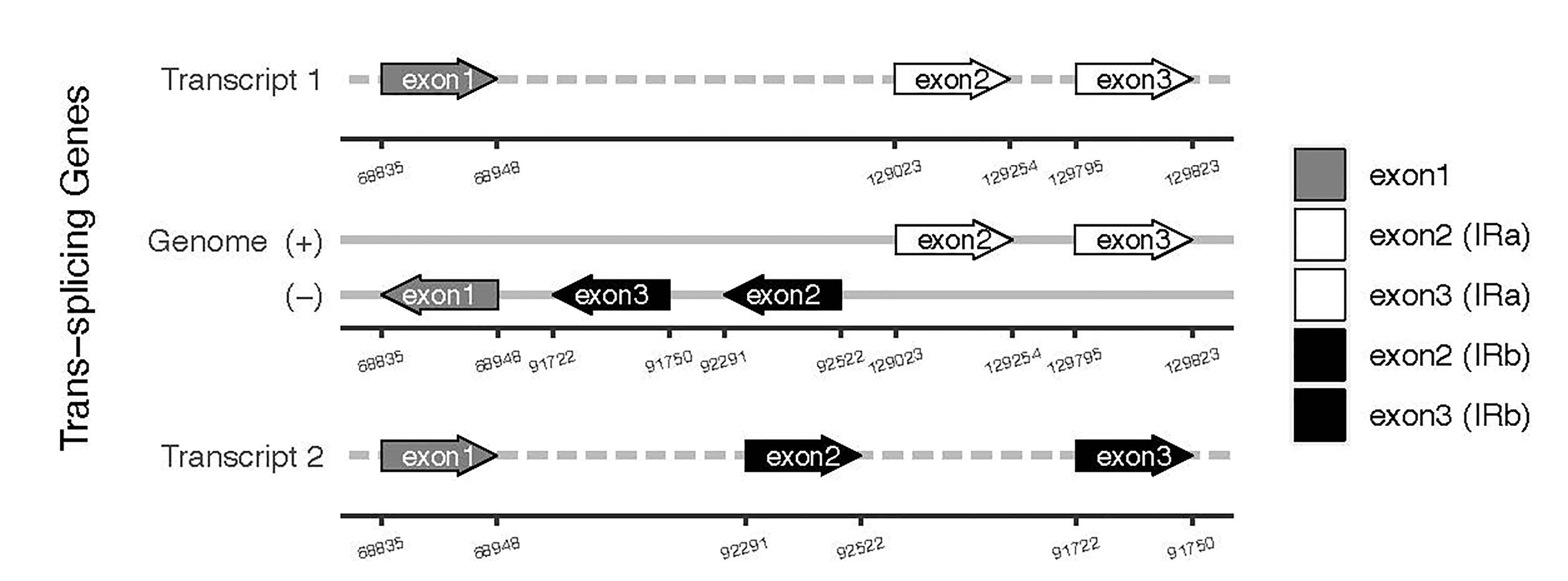

Supplement: Supplementary Figure 3.jpg [file TMDN_A_2519217_SM5723.jpg]
