# Supplementary material for: The complete chloroplast genome of eriochloa villosa (thunb.) kunth
Source: Mitochondrial DNA B Resour. 2025 Jun 24;10(7):637–40. doi: 10.1080/23802359.2025.2519217 (PMC12207762; doi:10.1080/23802359.2025.2519217)
Supplement: Supplymentary Figure legend.docx [file TMDN_A_2519217_SM5724.docx]

**Supplementary Figure 1.** Coverage depth distribution of the *Eriochloa villosa* chloroplast genome. The blue line indicates the sequencing depth at each genomic position.

**Supplementary Figure 2.** Structure of cis-splicing genes in the *Eriochloa villosa* chloroplast genome. The map of the cis-splicing genes, including nine cis-splicing genes (rps16, atpF, ycf3, petB, petD, rpl16, ndhB, ndhA, ndhB), eight of them have one intron and two exons, and one have two introns and three exons.

**Supplementary Figure 3.** Structure of trans-splicing genes in the *Eriochloa villosa* chloroplast genome. It has three exons，Two of which are duplicated as they are located in the IR regions. The genome sequence is shown in both positive (+) and negative (-) strands.
